# Genome-wide interaction analysis of menopausal hormone therapy use and breast cancer risk among 62,370 women

**DOI:** 10.1038/s41598-022-10121-2

**Published:** 2022-04-13

**Authors:** Xiaoliang Wang, Pooja Middha Kapoor, Paul L. Auer, Joe Dennis, Alison M. Dunning, Qin Wang, Michael Lush, Kyriaki Michailidou, Manjeet K. Bolla, Kristan J. Aronson, Rachel A. Murphy, Angela Brooks-Wilson, Derrick G. Lee, Emilie Cordina-Duverger, Pascal Guénel, Thérèse Truong, Claire Mulot, Lauren R. Teras, Alpa V. Patel, Laure Dossus, Rudolf Kaaks, Reiner Hoppe, Wing-Yee Lo, Thomas Brüning, Ute Hamann, Kamila Czene, Marike Gabrielson, Per Hall, Mikael Eriksson, Audrey Jung, Heiko Becher, Fergus J. Couch, Nicole L. Larson, Janet E. Olson, Kathryn J. Ruddy, Graham G. Giles, Robert J. MacInnis, Melissa C. Southey, Loic Le Marchand, Lynne R. Wilkens, Christopher A. Haiman, Håkan Olsson, Annelie Augustinsson, Ute Krüger, Philippe Wagner, Christopher Scott, Stacey J. Winham, Celine M. Vachon, Charles M. Perou, Andrew F. Olshan, Melissa A. Troester, David J. Hunter, Heather A. Eliassen, Rulla M. Tamimi, Kristen Brantley, Irene L. Andrulis, Jonine Figueroa, Stephen J. Chanock, Thomas U. Ahearn, Montserrat García-Closas, Gareth D. Evans, William G. Newman, Elke M. van Veen, Anthony Howell, Alicja Wolk, Niclas Håkansson, Hoda Anton-Culver, Argyrios Ziogas, Michael E. Jones, Nick Orr, Minouk J. Schoemaker, Anthony J. Swerdlow, Cari M. Kitahara, Martha Linet, Ross L. Prentice, Douglas F. Easton, Roger L. Milne, Peter Kraft, Jenny Chang-Claude, Sara Lindström

**Affiliations:** 1 Department of Epidemiology, School of Public Health, University of Washington, Seattle, WA USA; 2 Public Health Sciences Division, Fred Hutchinson Cancer Research Center, Seattle, WA USA; 3 Division of Cancer Epidemiology, German Cancer Research Center (DKFZ), Heidelberg, Germany; 4 University of Heidelberg, Faculty of Medicine, Heidelberg, Germany; 5 Cancer Prevention Program, Fred Hutchinson Cancer Research Center, Seattle, WA USA; 6 Division of Biostatistics, Institute for Health & Equity, and Cancer Center, Medical College of Wisconsin, Milwaukee, WI USA; 7 Department of Public Health and Primary Care, Centre for Cancer Genetic Epidemiology, University of Cambridge, Cambridge, UK; 8 Department of Oncology, Centre for Cancer Genetic Epidemiology, University of Cambridge, Cambridge, UK; 9 Biostatistics Unit, The Cyprus Institute of Neurology & Genetics, Nicosia, Cyprus; 10 The Cyprus Institute of Neurology & Genetics, Cyprus School of Molecular Medicine, Nicosia, Cyprus; 11 Department of Public Health Sciences, Cancer Research Institute, Queen’s University, Kingston, ON Canada; 12 School of Population and Public Health, University of British Columbia, Vancouver, BC Canada; 13 BC Cancer, Cancer Control Research, Vancouver, BC Canada; 14 Genome Sciences Centre, BC Cancer Agency, Vancouver, BC Canada; 15 Department of Biomedical Physiology and Kinesiology, Simon Fraser University, Burnaby, BC Canada; 16 Department of Mathematics and Statistics, St. Francis Xavier University, Antigonish, NS Canada; 17 Team Exposome and Heredity, Center for Research in Epidemiology and Population Health (CESP), INSERM, University Paris-Saclay, Villejuif, France; 18 INSERM UMR-S1147, Université Paris Sorbonné, Paris, France; 19 Department of Population Science, American Cancer Society, Atlanta, GA USA; 20 Nutrition and Metabolism Branch, International Agency for Research on Cancer (IARC-WHO), Lyon, France; 21 Dr. Margarete Fischer-Bosch-Institute of Clinical Pharmacology, Stuttgart, Germany; 22 University of Tÿbingen, Tÿbingen, Germany; 23 Institute for Prevention and Occupational Medicine of the German Social Accident Insurance, Institute of the Ruhr University Bochum (IPA), Bochum, Germany; 24 Molecular Genetics of Breast Cancer, German Cancer Research Center (DKFZ), Heidelberg, Germany; 25 Department of Medical Epidemiology and Biostatistics, Karolinska Institutet, Stockholm, Sweden; 26 Department of Oncology, Södersjukhuset, Stockholm, Sweden; 27 Institute of Medical Biometry and Epidemiology, University Medical Center Hamburg-Eppendorf, Hamburg, Germany; 28 Department of Laboratory Medicine and Pathology, Mayo Clinic, Rochester, MN USA; 29 Department of Health Sciences Research, Mayo Clinic, Rochester, MN USA; 30 Department of Oncology, Mayo Clinic, Rochester, MN USA; 31 Cancer Epidemiology Division, Cancer Council Victoria, Melbourne, VIC Australia; 32 Centre for Epidemiology and Biostatistics, Melbourne School of Population and Global Health, The University of Melbourne, Melbourne, VIC Australia; 33 Precision Medicine, School of Clinical Sciences at Monash Health, Monash University, Clayton, VIC Australia; 34 Department of Clinical Pathology, The University of Melbourne, Melbourne, VIC Australia; 35 Epidemiology Program, University of Hawaii Cancer Center, Honolulu, HI USA; 36 Cancer Epidemiology Program, University of Hawaii Cancer Center, Honolulu, HI USA; 37 Department of Preventive Medicine, Keck School of Medicine, University of Southern California, Los Angeles, CA USA; 38 Clinical Sciences, Department of Cancer Epidemiology, Lund University, Lund, Sweden; 39 Division of Biomedical Statistics and Informatics, Department of Health Sciences Research, Mayo Clinic, Rochester, MN USA; 40 Division of Epidemiology, Department of Health Science Research, Mayo Clinic, Rochester, MN USA; 41 Department of Genetics, Lineberger Comprehensive Cancer Center, University of North Carolina at Chapel Hill, Chapel Hill, NC USA; 42 Department of Epidemiology, Gillings School of Global Public Health, UNC Lineberger Comprehensive Cancer Center, University of North Carolina at Chapel Hill, Chapel Hill, NC USA; 43 Nuffield Department of Population Health, University of Oxford, Oxford, UK; 44 Department of Epidemiology, Harvard T.H. Chan School of Public Health, Boston, MA USA; 45 Channing Division of Network Medicine, Department of Medicine, Brigham and Women’s Hospital, Harvard Medical School, Boston, MA USA; 46 Department of Population Health Sciences, Weill Cornell Medicine, New York, NY USA; 47 Lunenfeld-Tanenbaum Research Institute of Mount Sinai Hospital, Fred A. Litwin Center for Cancer Genetics, Toronto, ON Canada; 48 Department of Molecular Genetics, University of Toronto, Toronto, ON Canada; 49 Usher Institute of Population Health Sciences and Informatics, The University of Edinburgh, Edinburgh, UK; 50 Cancer Research UK Edinburgh Centre, The University of Edinburgh, Edinburgh, UK; 51 Division of Cancer Epidemiology and Genetics, Department of Health and Human Services, National Cancer Institute, National Institutes of Health, Bethesda, MD USA; 52 Division of Evolution and Genomic Sciences, School of Biological Sciences, Faculty of Biology, Medicine and Health, Manchester Academic Health Science Centre, University of Manchester, Manchester, UK; 53 North West Genomics Laboratory Hub, Manchester Centre for Genomic Medicine, Manchester Academic Health Science Centre, St Mary’s Hospital, Manchester University NHS Foundation Trust, Manchester, UK; 54 Division of Cancer Sciences, University of Manchester, Manchester, UK; 55 Institute of Environmental Medicine, Karolinska Institutet, Stockholm, Sweden; 56 Department of Surgical Sciences, Uppsala University, Uppsala, Sweden; 57 Department of Medicine, Genetic Epidemiology Research Institute, University of California Irvine, Irvine, CA USA; 58 Division of Genetics and Epidemiology, The Institute of Cancer Research, London, UK; 59 Centre for Cancer Research and Cell Biology, Queen’s University Belfast, Belfast, Ireland UK; 60 Division of Breast Cancer Research, The Institute of Cancer Research, London, UK; 61 Radiation Epidemiology Branch, Division of Cancer Epidemiology and Genetics, National Cancer Institute, Bethesda, MD USA; 62 Program in Genetic Epidemiology and Statistical Genetics, Harvard T.H. Chan School of Public Health, Boston, MA USA; 63 Cancer Epidemiology Group, University Medical Center Hamburg-Eppendorf, University Cancer Center Hamburg (UCCH), Hamburg, Germany

**Keywords:** Genome-wide association studies, Risk factors, Breast cancer, Cancer epidemiology

## Abstract

Use of menopausal hormone therapy (MHT) is associated with increased risk for breast cancer. However, the relevant mechanisms and its interaction with genetic variants are not fully understood. We conducted a genome-wide interaction analysis between MHT use and genetic variants for breast cancer risk in 27,585 cases and 34,785 controls from 26 observational studies. All women were post-menopausal and of European ancestry. Multivariable logistic regression models were used to test for multiplicative interactions between genetic variants and current MHT use. We considered interaction p-values < 5 × 10^–8^ as genome-wide significant, and p-values < 1 × 10^–5^ as suggestive. Linkage disequilibrium (LD)-based clumping was performed to identify independent candidate variants. None of the 9.7 million genetic variants tested for interactions with MHT use reached genome-wide significance. Only 213 variants, representing 18 independent loci, had p-values < 1 × 10^5^. The strongest evidence was found for rs4674019 (p-value = 2.27 × 10^–7^), which showed genome-wide significant interaction (p-value = 3.8 × 10^–8^) with current MHT use when analysis was restricted to population-based studies only. Limiting the analyses to combined estrogen–progesterone MHT use only or to estrogen receptor (ER) positive cases did not identify any genome-wide significant evidence of interactions. In this large genome-wide SNP-MHT interaction study of breast cancer, we found no strong support for common genetic variants modifying the effect of MHT on breast cancer risk. These results suggest that common genetic variation has limited impact on the observed MHT–breast cancer risk association.

## Introduction

Breast cancer is one of the most common cancers in women. There were 268,600 new cases and 41,760 deaths due to breast cancer estimated in the U.S. in 2019^1^. The use of menopausal hormone therapy (MHT) is associated with up to 23% increased risk of breast cancer. MHT use has been reduced among postmenopausal women since the report by the Women’s Health Initiative (WHI) clinical trial and observational study^2,3^ which has been subsequently confirmed by other studies and meta-analyses^4,5^. Breast cancer risk increases with longer duration of use^6^, and is higher for combined estrogen–progesterone MHT (EPT) use as compared with estrogen-only (ET) regimens^4,5^. Additionally, the association between MHT use and breast cancer may also differ by tumor molecular subtype. A prospective cohort study in UK found that current MHT use was associated with increased risk for estrogen receptor positive (ER+) breast cancers, but not with ER- breast cancers^7^. Several other observational studies also found that MHT use was associated with elevated risk of ER+ breast cancer^8–12^.

The biological mechanisms underlying the effect of MHT use on breast cancer risk is not fully understood. One proposed mechanism is that higher estrogen and progesterone levels increase the proliferation of breast epithelial cells, which results in accumulation of genetic mutations and insufficient DNA repair^13,14^, and therefore induces mutagenesis^15,16^. Genome-wide association studies (GWAS) have identified over 200 single nucleotide polymorphisms (SNPs) that are associated with invasive breast cancer risk^17–19^. Further analyses based on these GWAS findings have identified several genes that might interact with MHT use on breast cancer risk, including SNPs regulating the fibroblast growth factor receptor two (*FGFR2*) gene^20^, as well as SNPs close to the Kruppel like factor 4 *(KLF4*) gene and the insulin like growth-factor-binding protein 5 (*IGFBP5*) gene^21–23^. A meta-analysis of four genome-wide case-only interaction studies found suggestive evidence of interactions between MHT use and SNPs in genes related to transmembrane signaling and immune cell activation^24^. However, none of the findings reached genome-wide significance.

In the present study, we performed a comprehensive genome-wide interaction analysis of current MHT use by pooling individual-level data from 26 epidemiological studies. We also performed genome-wide interaction analysis of MHT use on ER+ breast cancer specifically.

## Methods

### Study population and data collection

Individual level data were pooled from 26 epidemiological studies, including eight population-based case–control studies, 13 nested studies from prospective cohort studies and five studies with mixed design from the Breast Cancer Association Consortium (BCAC) (Table S1). Data collection instruments for individual studies have been described previously^19,23^. Breast cancer cases were defined as incident invasive or in-situ breast tumors, confirmed by medical records, pathological reports or death certificates. Cases of benign breast disease or cases diagnosed more than five years before study enrollment were excluded.

Participants were excluded if they were male, pre-menopausal, of non-European ancestry, with unknown age at reference date, or missing information on MHT use. Reference date was defined as date of diagnosis for cases, and date of interview for controls. Menopausal status was reported at time of interview. For women with missing menopausal status, we assumed postmenopausal status for those who were > 54 years old. Only studies with information on MHT use in at least 150 breast cancer cases and 150 controls were included in the data analysis.

### Ethnical approval and consent to participate

All participating studies were approved by the relevant ethics committees and informed consent was obtained from study participants.

### Menopausal hormone therapy use definition

MHT use was defined as use for at least three months of any type of MHT, including EPT and ET. Current MHT use was defined as use at, or within the six months prior to the reference date. Former MHT use was defined as women who had a history of using MHT but had quit more than 6 months prior to the reference date.

### Genotyping

Samples were genotyped by the Illumina custom iSelect genotyping array (iCOGs)^25,26^ or the Illumina OncoArray 500K (OncoArray)^19,27^. Details on genotyping, imputation and quality-control checks have been published previously^19,26^. For these analyses, 9680 cases and 10,598 controls were genotyped using iCOGs, and 17,905 cases and 24,187 controls were genotyped using OncoArray. Both datasets were imputed to the 1000 Genomes Phase 3 release^28^. For samples that were genotyped on both iCOGs and OncoArray, OncoArray data was used. SNPs were excluded if imputation r^2^ < 0.5 for iCOGs, and r^2^ < 0.8 for OncoArray. A total of 9,661,037 genetic variants (SNPs and indels) were included for analysis in both datasets.

### Statistical analysis

We used multivariable logistic regression models to test for interaction between each genetic variant and current MHT use (compared to never users) on breast cancer risk, adjusting for age at reference date, study, former MHT use, an indicator for study design (1 for population-based case–control or prospective studies, 0 for non-population or mixed case–control studies), an interaction term of study design indicator and current MHT use to account for different main effect of current MHT use by study design, and principal components to account for potential population stratification^29^, thus fitting a model of the form:

$$\begin{aligned} & logit\left[\mathrm{Pr}\left(D\right)\right]= \alpha +{\beta }_{c}CurrentMHT+{\beta }_{g}SNP+{\beta }_{gc}SNP\times CurrentMHT\\&\quad\quad\quad\quad\quad\quad\quad+{\beta }_{a}age+\sum_{i}{\beta }_{i}{study}_{i}+{\beta }_{f}FormerMHT+{\beta }_{p}PopulationBased \\&\quad\quad\quad\quad\quad\quad\quad+{\beta }_{pc}PopulationBased\times CurrentMHT+\sum_{j}{\beta }_{j}{PC}_{j} \end{aligned}$$

Each genetic variant was assessed as a continuous variable in a log-additive odds ratio model. For genetic variants that were not directly genotyped, the expected number of copies of the variant allele (“dosage”) was used^30^. OncoArray and iCOGs datasets were analyzed separately, and platform-specific interaction parameter estimates (${\beta }_{gc}$) were combined using METAL^31^ to obtain summary estimates for each SNP. Similar analyses were also performed for EPT use only and for ER+ breast cancer. Q-Q plots were used to assess whether the distribution of the p-values indicated genomic inflation. A p-value at 5 × 10^–8^ was used as the genome-wide significance level^32^.

For variants reaching suggestive evidence of interaction (p < 1 × 10^–5^), we performed linkage disequilibrium (LD)-based clumping to identify independent loci that might interact with MHT use on breast cancer risk (SWISS version 1.0.05b). SNPs in LD (r^2^ > 0.1 based on the build-in 1000G_2014-11_EUR) within 1 Mb from the most significantly associated SNP were removed so that independent SNPs remained in each region.

We also performed sensitivity analysis among patients from the population-based studies only. All analyses were performed using R version 3.6.1 unless otherwise specified.

## Results

**Figure 1:**
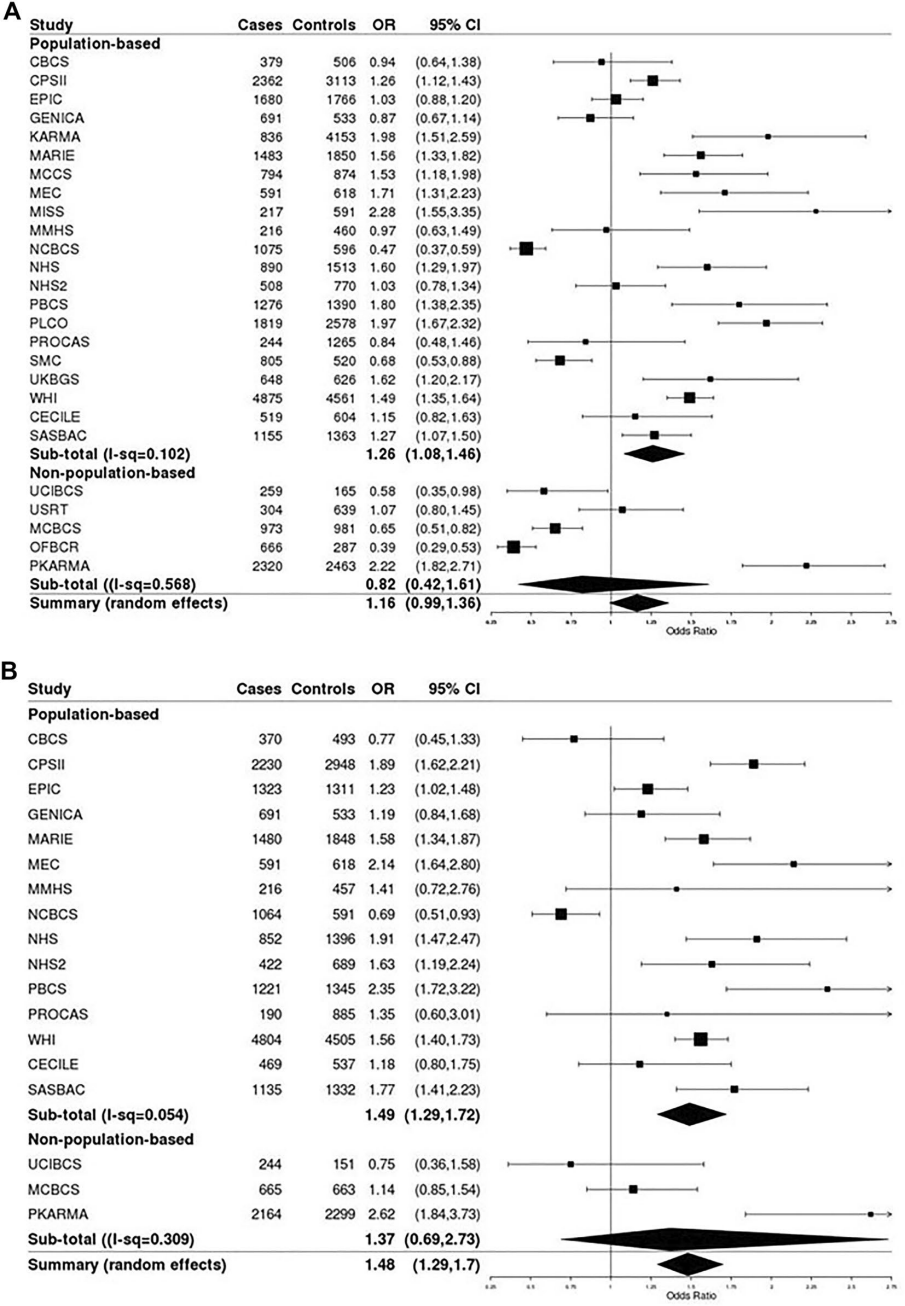
Main effects of current menopausal hormone therapy use and breast cancer risk by study. **(A)** Current use of any menopausal hormone therapy. **(B)** Current use of combined estrogen–progesterone menopausal hormone therapy.

A total of 62,370 post-menopausal women from 26 studies (27,585 cases and 34,785 controls), were included in the analyses (Table S1). Cases were slightly older (mean age: 64 years) than controls (mean age: 63 years). Current use of MHT was more common among breast cancer cases (34%) than controls (28%), showing a suggestive increased breast cancer risk (OR = 1.16; 95% CI: 0.99, 1.36; Fig. 1A). A total of 20,131 cases and 22,601 controls from 18 studies also had information on current use of EPT. Current EPT use was more common among cases (19%) than controls (13%) and was associated with an estimated 48% risk increase of breast cancer, compared to non-EPT users (OR: 1.48; 95% CI: 1.29, 1.70; Fig. 1B).

**Figure 2:**
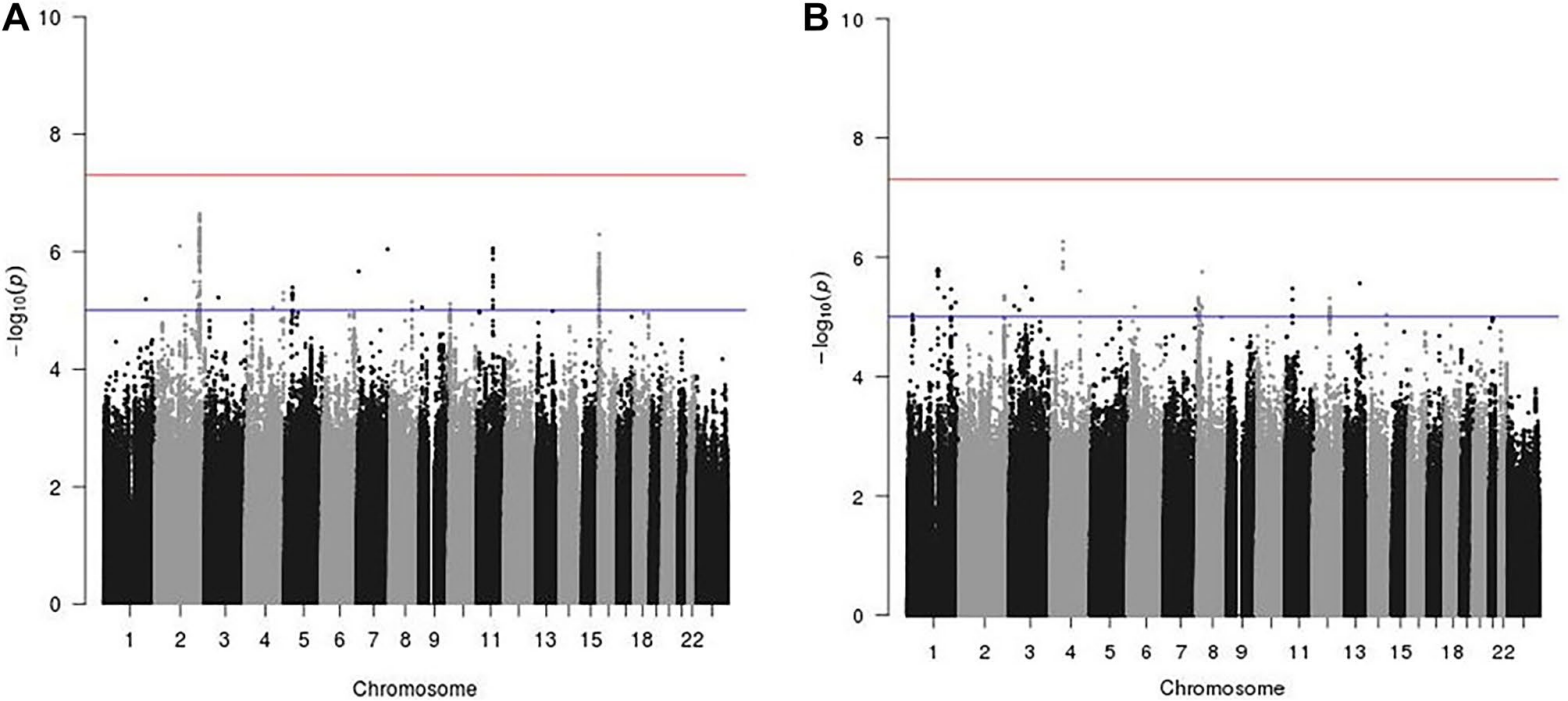
Manhattan plot of genome-wide interaction of current use of menopausal hormone therapy on breast cancer risk. **(A)** Current MHT use. **(B)** Current EPT use. Asterisk: red line: log-transformed genome-wide significant threshold at 5 × 10^–8^; blue line: log-transformed suggestive threshold at 1 × 10^–5^.

A total of 9,661,271 SNPs and indels were successfully imputed from both the OncoArray and iCOGs genotyping platforms and were included in the combined analysis. We did not observe any interactions between variants and current MHT use at genome-wide significance level (p-value < 5 × 10^–8^, Fig. 2A). 213 SNPs had suggestive evidence of interaction with MHT use on breast cancer risk (p-value < 1 × 10^–5^). After LD-based clumping, 18 independent SNPs remained, none of which were in LD with currently known breast cancer risk GWAS loci (Table 1). The strongest evidence of interaction was for SNP rs4674019, located at chromosome 2q35 (p-value = 2.27 × 10^–7^). When restricting the analyses to population-based studies only (23,063 cases and 30,250 controls), this same SNP rs4674019 showed statistically significantly interaction with current MHT use on breast cancer risk (p-value = 3.75 × 10^–8^; Fig. S1).

**Table 1:** Independent genetic variants with suggestive interactions of current MHT use on breast cancer risk after LD-based clumping.

SNP rsid^a^	Chr	Position^a^	A1	A2	EAF	Nearby genes	OR	95% CI	P-value
rs4674019	2	216601295	A	G	0.05	*LINC00607*	0.74	(0.66, 0.83)	2.27E−07
rs12600110	16	962154	T	C	0.38	*LMF1*	1.14	(1.08, 1.20)	5.11E−07
rs548302406	2	120097151	T	TA	0.35	*C2orf76*	0.86	(0.81, 0.92)	8.03E−07
rs117199302	11	77378563	T	C	0.02	*RSF1*	0.60	(0.49, 0.73)	8.82E−07
rs150004705	7	147063496	A	G	0.01	*CNTNAP2*	0.57	(0.46, 0.72)	9.13E−07
rs188419699	7	6674441	G	A	0.99	*ZNF853*	0.40	(0.27, 0.58)	2.16E−06
rs12600110	2	189608701	C	T	0.99	*DIRC1, LOC105373790*	0.48	(0.36, 0.66)	3.26E−06
rs11738429	5	36167878	G	A	0.19	*SKP2*	1.17	(1.09, 1.25)	4.09E−06
rs13121484	4	182999291	A	G	0.33	*AC108142.1*	1.14	(1.08, 1.21)	5.02E−06
rs74617030	2	206040628	G	GA	0.55	*PARD3B*	1.13	(1.07, 1.19)	5.90E−06
rs146251672	3	64290001	G	C	0.98	*PRICKLE2, LRRN1*	1.50	(1.26, 1.78)	6.08E−06
rs560643086	1	204318668	C	CA	0.75	*PLEKHA6*	1.16	(1.09, 1.24)	6.46E−06
rs79001083	8	106538183	A	C	0.05	*ZFPM2*	1.30	(1.16, 1.46)	7.12E−06
rs7900145	10	4933685	T	G	0.24	*AKR1C6P*	1.15	(1.08, 1.22)	7.75E−06
rs375101296	11	78180810	C	CAG	0.94	*NARS2*	0.77	(0.69, 0.86)	8.23E−06
rs72692777	9	10011536	T	C	0.02	*PTPRD*	0.70	(0.59, 0.82)	8.86E−06
rs142227065	4	132518511	T	TA	0.001	*RP11-314N14.1*	0.22	(0.11, 0.43)	9.07E−06
rs10015072	4	31386277	T	C	0.836	*RP11-315A17.1*	1.18	(1.10, 1.27)	9.67E−06

*Chr* chromosome, *A1* reference allele, *A2* alternative allele, *EAF* estimated allele frequency for alternative allele, *OR* odds ratios error per alternative allele with current menopausal hormone therapy use on breast cancer risk, *95% CI* corresponding 95% confidence intervals.

^a^rsid and position are based on the Genome Reference Consortium Human genome build 37.

Similarly, we did not observe any genome-wide significant interactions between SNPs and combined EPT use on breast cancer risk (Fig. 2B). There were 71 SNPs that reached suggestive significance level at p-value < 1 × 10^–5^. After LD-based clumping, 21 independent SNPs showed suggestive interactions (Table 2). The strongest evidence of interaction was for SNP rs4865075, located on chromosome 4q12 (p-value = 5.5 × 10^–7^). Sensitivity analysis using population-based studies only did not find statistically significant interactions.

**Table 2:** Independent genetic variants with suggestive interaction of current combined EPT use on breast cancer after LD-based clumping.

SNP rsid^a^	Chr	Position^a^	A1	A2	EAF	Nearby genes	OR	95% CI	P-value
rs4865075	4	57113130	A	G	0.76	*KIAA1211*	1.25	(1.14, 1.36)	5.50E−07
rs7519793	1	147301176	C	T	0.40	*RP11-433J22.3, RP11-314N2.2*	1.21	(1.12, 1.31)	1.59E−06
rs4871847	8	22964316	A	G	0.30	*TNFRSF10C*	0.82	(0.85, 0.89)	1.77E−06
rs2165698	13	88558039	T	C	0.54	*TET1P1, RP11-545P6.2*	1.22	(1.12, 1.33)	2.76E−06
rs34954573	3	76118773	CT	C	0.56	*ROBO2*	0.82	(0.76, 0.89)	3.17E−06
rs10836138	11	33996495	C	T	0.47	*LMO2, CAPRIN1*	0.83	(0.77, 0.90)	3.37E−06
rs4844958	1	210361388	A	G	0.47	*SYT14, SERTAD4-AS1*	1.20	(1.11, 1.30)	3.45E−06
rs145119792	4	138681096	G	T	0.99	*RP13-884E18.4, RP11-793B23.1*	2.61	(1.74, 3.91)	3.71E−06
rs2372593	2	216596263	G	A	0.93	*LINC00607*	1.42	(1.22, 1.66)	4.52E−06
rs1359939	1	177820861	G	A	0.68	*RP11-63B19.1, SEC16B*	1.22	(1.12, 1.33)	4.71E−06
rs1398476	8	5608189	C	A	0.79	*RP11-281H11.1, RP11-728L1.1*	0.79	(0.81, 0.87)	4.81E−06
rs148904951	12	78267629	T	G	0.03	*NAV3*	2.05	(1.51, 2.78)	4.90E−06
rs41380949	3	105175646	A	G	0.10	*ALCAM*	1.36	(1.19, 1.55)	5.11E−06
rs116807456	1	232593292	A	G	0.02	*SIPA1L2*	0.49	(0.36, 0.66)	5.76E−06
rs79505632	8	11079796	C	G	0.05	*AF131215.8, LINC00529*	0.66	(0.55, 0.79)	6.17E−06
rs146727380	3	21750977	GAAAAC	GAAAACAAAAC	0.51	*ZNF385D*	0.82	(0.76, 0.90)	6.61E−06
rs146444598	6	29912227	TGGA	T	0.52	*HLA-A*	1.19	(1.10, 1.29)	6.85E−06
rs9690705	7	151595436	G	A	0.42	*PRKAG2-AS1, RNU6-604P*	1.20	(1.11, 1.29)	7.46E−06
rs77773073	3	44919409	G	A	0.86	*TGM4, LRRN1*	1.28	(1.15, 1.43)	7.65E−06
rs1772028	14	101693861	C	G	0.49	*RP11-8L8.1, CTD-2561F5.1*	0.84	(0.78, 0.91)	9.16E−06
rs7522223	1	25217994	T	C	0.15	*CLIC4, RUNX3*	0.78	(0.70, 0.87)	9.26E−06

*SNP* single nucleotide polymorphism, *Chr* chromosome, *A1* reference allele, *A2* alternative allele, *EAF* estimated allele frequency for alternative allele, *OR* odds ratios per alternative allele with current combined estrogen–progesterone hormone therapy use on breast cancer risk, *95% CI* corresponding 95% confidence intervals;

^a^rsid and position are based on the Genome Reference Consortium Human genome build 37.

Restricting our cases to those with ER+ breast cancer did not result in any genome-wide significant findings (Figs. S2 and S3). No genomic inflation was observed in primary or subgroup analyses (Figs. S1, S2 and S3).

## Discussion

In this large genome-wide analysis of postmenopausal women of European ancestry, we did not identify any genetic variants that were strong modifiers of the association between current MHT use on breast cancer risk. Although the interaction between SNP rs4674019 and current MHT use was statistically significant among population-based studies only, the variate allele frequency is relatively rare (EAF = 5%) and needs further validation.

Consistent with previous literature^2,3,33^, we found that current use of MHT, and in particular current EPT use, was associated with an increased risk of breast cancer for postmenopausal women. The mechanisms underlying this association are not fully understood. It has been hypothesized that estrogen stimulates cell proliferation through ERα-mediated hormone activity and increases mutation rates through a cytochrome P450-mediated metabolic activation that results in DNA damage^34^. In addition, the risk associated with ER+ breast cancer is substantially higher than for ER- breast cancer, particularly for EPT use, suggesting an ER-dependent pathway^5^. In vitro and in vivo studies found that estradiol and 4-OH-estradiol, metabolites of estrogen, may induce mutations and damage DNA by forming DNA adducts to bind to adenine and guanine on the DNA backbone^35,36^. The role of progestogens in human breast carcinogenesis is less clear, although it has been suggested that synthetic progestogens are pro-proliferative and may thus promote cancer cell growth^37,38^.

Although MHT use has been found to be associated with increased breast cancer risk in both epidemiologic and experimental studies, no published studies to date have identified genome-wide significant interactions for breast cancer risk between candidate single variants and MHT use among postmenopausal women^39,40^. In a previous two-stage GWAS interaction analysis among ~ 2700 cases and ~ 2700 controls, five SNPs had suggestive evidence of interaction with current MHT use; but none of them reached genome-wide significance^41^. A meta-analysis of genome-wide case-only studies in 2920 cases also found no statistically significant interactions between SNPs and MHT use on breast cancer overall or by subtype^24^. Although our study had a larger sample size and increased statistical power than previous genome-wide analyses, we similarly did not find any genome-wide statistically significant interactions between genetic variants and MHT use in this study, and we further did not replicate previously suggested SNPs (data not shown).

The region for which the strongest evidence of interaction with current MHT use on breast cancer risk was observed (lead SNP rs4674019), was also implicated in the analysis restricted to combined EPT use only (p-value = 4.5 × 10^–6^). The rs4674019 SNP is an intronic variant in the coding region for the long intergenic non-protein coding RNA 607 (*LINC00607*). Although the functionality of long non-coding RNAs is still not clear, it has been recently recognized that abnormal expression of long non-coding RNAs may play an important role in cell cycle control and cell differentiation, which is related to cancer and neurodegenerative disease^42–44^. Expression levels of LINC00607 were found to be significantly downregulated among lung adenocarcinoma tissues, compared to adjacent tissues^45^. Other GWAS have shown genetic variants in the *LINC00607* gene to be associated with height in people of European ancestry^46^. Previous evidence for long noncoding RNAs in relation to breast cancer risk is limited; but it is possible that changes in exogenous hormone levels due to MHT use result in differential expression that eventually leads to tumorigenesis.

We also observed suggestive evidence of interaction between current use of both MHT and EPT and rs146251672. SNP rs146251672 is located in the intronic region for the prickle planar cell polarity protein 2 (*PRICKLE2*) gene on chromosome 3. *PRICKLE2* encodes a non-canonical *Wnt* signaling protein that mediates feedback amplification to generate asymmetric planar cell polarity (PCP) signaling^47^. The Wnt pathway has been found to be activated in more than half of breast tumors, and is associated with lower overall survival for breast cancer patients^48^. In particular, the upregulation of the Wnt/PCP pathway has been suggested to be associated with more malignant phenotypes, such as abnormal tissue polarity, invasion and metastasis^49^. Exposure to estrogen has been associated with accelerated tumor formation in ER-knockout/Wnt-1 mice^36^. It is plausible that MHT acts partially through the alternative *Wnt* pathway rather than ER-dependent pathways to promote breast tumor development.

This study constitutes the largest genome-wide interaction analysis for current MHT use and breast cancer risk in postmenopausal women to date. We analyzed data from more than 62,000 women for whom we had both MHT use and genotypes from more than 9.6 million genetic variants. We controlled our analysis for potential confounding by population stratification by adjusting for principal components. We performed LD-based clumping, which accounted for correlations between genotypes to identify the strongest signal in each independent region, providing more targeted variants and regions for future investigation.

There are some limitations to our study. We used a single binary definition of current MHT use within 6 months prior to reference date and could not evaluate other measures such as age at MHT initiation or duration of MHT use. This could lead to some exposure misclassification, particularly for the non-population based studies, where it is possible that those cases had stopped their MHT use at time of recruitment and were classified as non-current users. Such misclassification would have attenuated the main effect of MHT and reduced our statistical power to detect any interactions. In our sensitivity analysis using population-based studies only, we found stronger interactions between the lead SNPs and MHT use. However, given a smaller sample size in the sensitivity analysis, it is possible that we did not have sufficient statistical power to detect any other potential interactions. We assumed no significant interactions between SNPs and former MHT use, and only adjusted for potential confounding from the main effect of former MHT use in the model based on previous evidence. It is possible that comparing the interaction effect of current MHT use to a combined reference group of never and former users may attenuate the point estimates of the interaction term. The use of estrogen only hormone therapy (ET) was also not available among the study participants, although the statistical power might be further limited since the main association of ET and breast cancer risk is much smaller than EPT use^5^. Although the impact may be small given our case–control study design and large sample size, it is still possible that the observed SNP-MHT interaction was due to the interaction between SNPs and potential uncontrolled confounders of MHT use that were not available in our study^50^, such as tolerance of menopausal symptoms or socioeconomic status. In addition, our study sample only included women of European ancestry, and thus, our findings may not be generalizable to other race/ethnicity groups.

It is important to note that the lack of statistical interaction, on the log-scale, does not necessarily imply a lack of biological interaction. The results are consistent with a model in which the effects of genetic variants and MHT use combine multiplicatively on risk, which could still indicate important interactions at a functional level. Overall, our results suggest that it is not necessary to include interaction variables for G × MHT use in development of breast cancer risk prediction models. Although our results suggested that potential interaction effect between SNP rs4674019 and current MHT, further validation is needed. Several suggestive interactions also warrant further investigations in independent studies.

## Conclusion

In this large genome-wide SNP-MHT interaction study of breast cancer, we found no strong support for common genetic variants modifying the effect of MHT on breast cancer risk. These results suggest that common genetic variation has limited impact on the observed MHT–breast cancer risk association.

## Supplementary Information

Supplementary Information.

## Data Availability

The data that support the findings of this study are available from the Breast Cancer Association Consortium but restrictions apply to the availability of these data, which were used under license for the current study, and so are not publicly available. Data are however available from the authors upon reasonable request and with permission of the Breast Cancer Association Consortium.

## Abbreviations

MHT: Menopausal hormone therapy
LD: Linkage disequilibrium
SNP: Single nucleotide polymorphism
EPT: Estrogen–progesterone menopausal hormone therapy
ET: Estrogen-only menopausal hormone therapy
ER+: Estrogen receptor positive
GWAS: Genome-wide association studies
BCAC: Breast Cancer Association Consortium
iCOGs: Samples genotyped using the Illumina custom iSelect genotyping array
OncoArray: Samples genotyped using the Illumina 500K array

## References

[1] Siegel RL, Miller KD, Jemal A (2019). Cancer statistics, 2019. CA Cancer J. Clin..

[2] Chlebowski RT, Kuller LH, Prentice RL, Stefanick ML, Manson JE, Gass M, Aragaki AK, Ockene JK, Lane DS, Sarto GE, Rajkovic A, Schenken R (2009). Breast cancer after use of estrogen plus progestin in postmenopausal women. N. Engl. J. Med..

[3] Prentice RL, Chlebowski RT, Stefanick ML, Manson JE, Langer RD, Pettinger M, Hendrix SL, Hubbell FA, Kooperberg C, Kuller LH, Lane DS, McTiernan A (2008). Conjugated equine estrogens and breast cancer risk in the Women's Health Initiative clinical trial and observational study. Am. J. Epidemiol..

[4] Anothaisintawee T, Wiratkapun C, Lerdsitthichai P, Kasamesup V, Wongwaisayawan S, Srinakarin J, Hirunpat S, Woodtichartpreecha P, Boonlikit S, Teerawattananon Y, Thakkinstian A (2013). Risk factors of breast cancer: A systematic review and meta-analysis. Asia Pac. J. Public Health.

[5] Collaborative Group on Hormonal Factors in Breast C. Type and timing of menopausal hormone therapy and breast cancer risk: Individual participant meta-analysis of the worldwide epidemiological evidence. *Lancet*. **394**, 1159–1168 (2019). 10.1016/S0140-6736(19)31709-X PMC6891893 31474332

[6] Breast cancer and hormone replacement therapy: Collaborative reanalysis of data from 51 epidemiological studies of 52,705 women with breast cancer and 108,411 women without breast cancer. Collaborative Group on Hormonal Factors in Breast Cancer. *Lancet* **350**, 1047–1059 (1997). 10213546

[7] Beral V, Reeves G, Bull D, Green J (2011). Million Women Study C. Breast cancer risk in relation to the interval between menopause and starting hormone therapy. J. Natl. Cancer Inst..

[8] Chen WY, Hankinson SE, Schnitt SJ, Rosner BA, Holmes MD, Colditz GA (2004). Association of hormone replacement therapy to estrogen and progesterone receptor status in invasive breast carcinoma. Cancer.

[9] Gertig DM, Fletcher AS, English DR, Macinnis RJ, Hopper JL, Giles GG (2006). Hormone therapy and breast cancer: What factors modify the association?. Menopause.

[10] Salagame U, Banks E, O'Connell DL, Egger S, Canfell K (2018). Menopausal hormone therapy use and breast cancer risk by receptor subtypes: Results from the New South Wales Cancer Lifestyle and EvaluAtion of Risk (CLEAR) study. PLoS ONE.

[11] Saxena T, Lee E, Henderson KD, Clarke CA, West D, Marshall SF, Deapen D, Bernstein L, Ursin G (2010). Menopausal hormone therapy and subsequent risk of specific invasive breast cancer subtypes in the California Teachers Study. Cancer Epidemiol. Biomarkers Prevent..

[12] Setiawan VW, Monroe KR, Wilkens LR, Kolonel LN, Pike MC, Henderson BE (2009). Breast cancer risk factors defined by estrogen and progesterone receptor status: The multiethnic cohort study. Am. J. Epidemiol..

[13] Mello ML, Vidal BC, Russo IH, Lareef MH, Russo J (2007). DNA content and chromatin texture of human breast epithelial cells transformed with 17-beta-estradiol and the estrogen antagonist ICI 182,780 as assessed by image analysis. Mutat. Res..

[14] Saeed M, Rogan E, Fernandez SV, Sheriff F, Russo J, Cavalieri E (2007). Formation of depurinating N3Adenine and N7Guanine adducts by MCF-10F cells cultured in the presence of 4-hydroxyestradiol. Int. J. Cancer.

[15] Persson I (2000). Estrogens in the causation of breast, endometrial and ovarian cancers—Evidence and hypotheses from epidemiological findings. J. Steroid Biochem. Mol. Biol..

[16] Yager JD, Liehr JG (1996). Molecular mechanisms of estrogen carcinogenesis. Annu. Rev. Pharmacol. Toxicol..

[17] Easton DF, Pooley KA, Dunning AM, Pharoah PD, Thompson D, Ballinger DG, Struewing JP, Morrison J, Field H, Luben R, Wareham N, Ahmed S (2007). Genome-wide association study identifies novel breast cancer susceptibility loci. Nature.

[18] Hunter DJ, Kraft P, Jacobs KB, Cox DG, Yeager M, Hankinson SE, Wacholder S, Wang Z, Welch R, Hutchinson A, Wang J, Yu K (2007). A genome-wide association study identifies alleles in FGFR2 associated with risk of sporadic postmenopausal breast cancer. Nat. Genet..

[19] Michailidou K, Lindstrom S, Dennis J, Beesley J, Hui S, Kar S, Lemacon A, Soucy P, Glubb D, Rostamianfar A, Bolla MK, Wang Q (2017). Association analysis identifies 65 new breast cancer risk loci. Nature.

[20] Prentice RL, Huang Y, Hinds DA, Peters U, Pettinger M, Cox DR, Beilharz E, Chlebowski RT, Rossouw JE, Caan B, Ballinger DG (2009). Variation in the FGFR2 gene and the effects of postmenopausal hormone therapy on invasive breast cancer. Cancer Epidemiol. Biomarkers Prevent..

[21] Ghoussaini M, Edwards SL, Michailidou K, Nord S, Cowper-Sal Lari R, Desai K, Kar S, Hillman KM, Kaufmann S, Glubb DM, Beesley J, Dennis J (2014). Evidence that breast cancer risk at the 2q35 locus is mediated through IGFBP5 regulation. Nat. Commun..

[22] Nickels S, Truong T, Hein R, Stevens K, Buck K, Behrens S, Eilber U, Schmidt M, Haberle L, Vrieling A, Gaudet M, Figueroa J (2013). Evidence of gene-environment interactions between common breast cancer susceptibility loci and established environmental risk factors. PLoS Genet..

[23] Kapoor PM, Lindstrom S, Behrens S, Wang X, Michailidou K, Bolla MK, Wang Q, Dennis J, Dunning AM, Pharoah PDP, Schmidt MK, Kraft P (2020). Assessment of interactions between 205 breast cancer susceptibility loci and 13 established risk factors in relation to breast cancer risk, in the Breast Cancer Association Consortium. Int. J. Epidemiol..

[24] Rudolph A, Hein R, Lindstrom S, Beckmann L, Behrens S, Liu J, Aschard H, Bolla MK, Wang J, Truong T, Cordina-Duverger E, Menegaux F (2013). Genetic modifiers of menopausal hormone replacement therapy and breast cancer risk: A genome-wide interaction study. Endocr. Relat. Cancer.

[25] Michailidou K, Beesley J, Lindstrom S, Canisius S, Dennis J, Lush MJ, Maranian MJ, Bolla MK, Wang Q, Shah M, Perkins BJ, Czene K (2015). Genome-wide association analysis of more than 120,000 individuals identifies 15 new susceptibility loci for breast cancer. Nat. Genet..

[26] Michailidou K, Hall P, Gonzalez-Neira A, Ghoussaini M, Dennis J, Milne RL, Schmidt MK, Chang-Claude J, Bojesen SE, Bolla MK, Wang Q, Dicks E (2013). Large-scale genotyping identifies 41 new loci associated with breast cancer risk. Nat Genet.

[27] Amos CI, Dennis J, Wang Z, Byun J, Schumacher FR, Gayther SA, Casey G, Hunter DJ, Sellers TA, Gruber SB, Dunning AM, Michailidou K (2017). The OncoArray Consortium: A network for understanding the genetic architecture of common cancers. Cancer Epidemiol. Biomarkers Prevent..

[28] Auton A, Brooks LD, Durbin RM, Garrison EP, Kang HM, Korbel JO, Marchini JL, McCarthy S, McVean GA, Abecasis GR, Genomes Project C (2015). A global reference for human genetic variation. Nature.

[29] Price AL, Patterson NJ, Plenge RM, Weinblatt ME, Shadick NA, Reich D (2006). Principal components analysis corrects for stratification in genome-wide association studies. Nat. Genet..

[30] Jiao S, Hsu L, Hutter CM, Peters U (2011). The use of imputed values in the meta-analysis of genome-wide association studies. Genet. Epidemiol..

[31] Willer CJ, Li Y, Abecasis GR (2010). METAL: Fast and efficient meta-analysis of genomewide association scans. Bioinformatics.

[32] Dudbridge F, Gusnanto A (2008). Estimation of significance thresholds for genomewide association scans. Genet. Epidemiol..

[33] Rossouw JE, Anderson GL, Prentice RL, LaCroix AZ, Kooperberg C, Stefanick ML, Jackson RD, Beresford SA, Howard BV, Johnson KC, Kotchen JM, Ockene J (2002). Risks and benefits of estrogen plus progestin in healthy postmenopausal women: Principal results From the Women's Health Initiative randomized controlled trial. JAMA.

[34] Russo J, Russo IH (2006). The role of estrogen in the initiation of breast cancer. J. Steroid Biochem. Mol. Biol..

[35] Cavalieri E, Chakravarti D, Guttenplan J, Hart E, Ingle J, Jankowiak R, Muti P, Rogan E, Russo J, Santen R, Sutter T (2006). Catechol estrogen quinones as initiators of breast and other human cancers: Implications for biomarkers of susceptibility and cancer prevention. Biochim. Biophys. Acta.

[36] Yue W, Wang JP, Li Y, Fan P, Liu G, Zhang N, Conaway M, Wang H, Korach KS, Bocchinfuso W, Santen R (2010). Effects of estrogen on breast cancer development: Role of estrogen receptor independent mechanisms. Int. J. Cancer.

[37] Horwitz KB, Sartorius CA (2008). Progestins in hormone replacement therapies reactivate cancer stem cells in women with preexisting breast cancers: A hypothesis. J. Clin. Endocrinol. Metab..

[38] Santen RJ (2003). Risk of breast cancer with progestins: Critical assessment of current data. Steroids.

[39] Harlid S, Butt S, Ivarsson MI, Eyfjord JE, Lenner P, Manjer J, Dillner J, Carlson J (2012). Interactive effect of genetic susceptibility with height, body mass index, and hormone replacement therapy on the risk of breast cancer. BMC Womens Health.

[40] Travis RC, Reeves GK, Green J, Bull D, Tipper SJ, Baker K, Beral V, Peto R, Bell J, Zelenika D, Lathrop M (2010). Million Women Study C. Gene-environment interactions in 7610 women with breast cancer: Prospective evidence from the Million Women Study. Lancet.

[41] Hein R, Flesch-Janys D, Dahmen N, Beckmann L, Lindstrom S, Schoof N, Czene K, Mittelstrass K, Illig T, Seibold P, Behrens S, Humphreys K (2013). A genome-wide association study to identify genetic susceptibility loci that modify ductal and lobular postmenopausal breast cancer risk associated with menopausal hormone therapy use: A two-stage design with replication. Breast Cancer Res. Treat..

[42] Dey BK, Mueller AC, Dutta A (2014). Long non-coding RNAs as emerging regulators of differentiation, development, and disease. Transcription..

[43] Ricciuti B, Mencaroni C, Paglialunga L, Paciullo F, Crino L, Chiari R, Metro G (2016). Long noncoding RNAs: New insights into non-small cell lung cancer biology, diagnosis and therapy. Med. Oncol..

[44] Zhao W, An Y, Liang Y, Xie XW (2014). Role of HOTAIR long noncoding RNA in metastatic progression of lung cancer. Eur. Rev. Med. Pharmacol. Sci..

[45] Zhao B, Xu H, Ai X, Adalat Y, Tong Y, Zhang J, Yang S (2018). Expression profiles of long noncoding RNAs in lung adenocarcinoma. Onco Targets Ther..

[46] Kichaev G, Bhatia G, Loh PR, Gazal S, Burch K, Freund MK, Schoech A, Pasaniuc B, Price AL (2019). Leveraging polygenic functional enrichment to improve GWAS power. Am. J. Hum. Genet..

[47] Tree DR, Shulman JM, Rousset R, Scott MP, Gubb D, Axelrod JD (2002). Prickle mediates feedback amplification to generate asymmetric planar cell polarity signaling. Cell.

[48] Khramtsov AI, Khramtsova GF, Tretiakova M, Huo D, Olopade OI, Goss KH (2010). Wnt/beta-catenin pathway activation is enriched in basal-like breast cancers and predicts poor outcome. Am. J. Pathol..

[49] Katoh M (2005). WNT/PCP signaling pathway and human cancer (review). Oncol. Rep..

[50] Vanderweele TJ, Ko YA, Mukherjee B (2013). Environmental confounding in gene-environment interaction studies. Am. J. Epidemiol..

