# Supplementary material for: Genome-wide interaction analysis of menopausal hormone therapy use and breast cancer risk among 62,370 women
Source: Sci Rep. 2022 Apr 13;12:6199. doi: 10.1038/s41598-022-10121-2 (PMC9007944; doi:10.1038/s41598-022-10121-2)
Supplement: Supplementary file 1 — Supplementary Information. [file 41598_2022_10121_MOESM1_ESM.pdf]

Supplemental Materials

Genome-wide interaction analysis of menopausal hormone therapy use and breast cancer risk among 62,370 women

Xiaoliang Wang[*,1,2], Pooja Middha Kapoor[3,4], Paul L. Auer[5,6], Joe Dennis[7], Alison M. Dunning[8], Qin Wang[7], Michael Lush[7], Kyriaki Michailidou[7,9,10], Manjeet K. Bolla[7], Kristan J. Aronson[11], Rachel A. Murphy[12,13], Angela Brooks-Wilson[14,15], Derrick G. Lee[13,16], Emilie Cordina-Duverger[17], Pascal Guénel[17], Thérèse Truong[17], Claire Mulot[18], Lauren R. Teras[19], Alpa V. Patel[19], Laure Dossus[20], Rudolf Kaaks[3], Reiner Hoppe[21,22], Wing-Yee Lo[21,22], Thomas Brüning[23], Ute Hamann[24], Kamila Czene[25], Marike Gabrielson[25], Per Hall[25,26], Mikael Eriksson[25], Audrey Jung[3], Heiko Becher[27], Fergus J. Couch[28], Nicole L. Larson[29], Janet E. Olson[29], Kathryn J. Ruddy[30], Graham G. Giles[31,32,33], Robert J. MacInnis[31,32], Melissa C. Southey[31,33,34], Loic Le Marchand[35], Lynne R. Wilkens[36], Christopher A. Haiman[37], Håkan Olsson[38], Annelie Augustinsson[38], Ute Krüger[38], Philippe Wagner[38], Christopher Scott[29], Stacey J. Winham[39], Celine M. Vachon[40], Charles M. Perou[40], Andrew F. Olshan[41], Melissa A. Troester[41], David J. Hunter[42,43], Heather A. Eliassen[43,44], Rulla M. Tamimi[43,45], Kristen Brantley[44], Irene L. Andrulis[46,47], Jonine Figueroa[48,49,50], Stephen J. Chanock[50], Thomas U. Ahearn[50], Montserrat García-Closas[50], Gareth D. Evans[51,52], William G. Newman[51,52], Elke M. van Veen[51,52], Anthony Howell[53], Alicja Wolk[54,55], Niclas Håkansson[54], Hoda Anton-Culver[56], Argyrios Ziogas[56], Michael E. Jones[57], Nick Orr[58], Minouk J. Schoemaker[57], Anthony J. Swerdlow[57,59], Cari M. Kitahara[60], Martha Linet[60], Ross L Prentice[2], Douglas F. Easton[7,8], Roger L. Milne[31,32,33], Peter Kraft[43,61], Jenny Chang-Claude[3,62], Sara Lindström[1,2]

* Corresponding author: Xiaoliang Wang; xwang23@fredhutch.org

Affiliations:

[1] University of Washington School of Public Health, Department of Epidemiology, Seattle, WA, USA.
[2] Fred Hutchinson Cancer Research Center, Public Health Sciences Division, Seattle, WA, USA.
[3] German Cancer Research Center (DKFZ), Division of Cancer Epidemiology, Heidelberg, Germany.
[4] University of Heidelberg, Faculty of Medicine, Heidelberg, Germany.
[5] Fred Hutchinson Cancer Research Center, Cancer Prevention Program, Seattle, WA, USA.
[6] University of Wisconsin-Milwaukee, Zilber School of Public Health, Milwaukee, WI, USA.
[7] University of Cambridge, Centre for Cancer Genetic Epidemiology, Department of Public Health and Primary Care, Cambridge, UK.
[8] University of Cambridge, Centre for Cancer Genetic Epidemiology, Department of Oncology, Cambridge, UK.
[9] The Cyprus Institute of Neurology & Genetics, Biostatistics Unit, Nicosia, Cyprus.
[10] The Cyprus Institute of Neurology & Genetics, Cyprus School of Molecular Medicine, Nicosia, Cyprus.
[11] Queen's University, Department of Public Health Sciences, and Cancer Research Institute, Kingston, ON, Canada.
[12] University of British Columbia, School of Population and Public Health, Vancouver, BC, Canada.
[13] BC Cancer, Cancer Control Research, Vancouver, BC, Canada.
[14] BC Cancer Agency, Genome Sciences Centre, Vancouver, BC, Canada.
[15] Simon Fraser University, Department of Biomedical Physiology and Kinesiology, Burnaby, BC, Canada.
[16] St. Francis Xavier University, Department of Mathematics and Statistics, Antigonish, NS, Canada.
[17] INSERM, University Paris-Saclay, Center for Research in Epidemiology and Population Health (CESP), Team Exposome and Heredity, Villejuif, France.
[18] INSERM UMR-S1147, Université Paris Sorbonné, Paris, France.

[19] American Cancer Society, Department of Population Science, Atlanta, GA, USA.
[20] International Agency for Research on Cancer (IARC-WHO), Nutrition and Metabolism Branch, Lyon, France.
[21] Dr. Margarete Fischer-Bosch-Institute of Clinical Pharmacology, Stuttgart, Germany.
[22] University of TŸbingen, TŸbingen, Germany.
[23] Institute of the Ruhr University Bochum (IPA), Institute for Prevention and Occupational Medicine of the German Social Accident Insurance, Bochum, Germany.
[24] German Cancer Research Center (DKFZ), Molecular Genetics of Breast Cancer, Heidelberg, Germany.

[25] Karolinska Institutet, Department of Medical Epidemiology and Biostatistics, Stockholm, Sweden.
[26] Södersjukhuset, Department of Oncology, Stockholm, Sweden.
[27] University Medical Center Hamburg-Eppendorf, Institute of Medical Biometry and Epidemiology, Hamburg, Germany.
[28] Mayo Clinic, Department of Laboratory Medicine and Pathology, Rochester, MN, USA.
[29] Mayo Clinic, Department of Health Sciences Research, Rochester, MN, USA.
[30] Mayo Clinic, Department of Oncology, Rochester, MN, USA.
[31] Cancer Council Victoria, Cancer Epidemiology Division, Melbourne, Victoria, Australia.
[32] The University of Melbourne, Centre for Epidemiology and Biostatistics, Melbourne School of Population and Global Health, Melbourne, Victoria, Australia.
[33] Monash University, Precision Medicine, School of Clinical Sciences at Monash Health, Clayton, Victoria, Australia.
[34] The University of Melbourne, Department of Clinical Pathology, Melbourne, Victoria, Australia.
[35] University of Hawaii Cancer Center, Epidemiology Program, Honolulu, HI, USA.
[36] University of Hawaii Cancer Center, Cancer Epidemiology Program, Honolulu, HI, USA.
[37] University of Southern California, Department of Preventive Medicine, Keck School of Medicine, Los Angeles, CA, USA.
[38] Lund University, Department of Cancer Epidemiology, Clinical Sciences, Lund, Sweden.
[39] Mayo Clinic, Department of Health Sciences Research, Division of Biomedical Statistics and Informatics, Rochester, MN, USA.
[40] Mayo Clinic, Department of Health Science Research, Division of Epidemiology, Rochester, MN, USA.
[40] University of North Carolina at Chapel Hill, Department of Genetics, Lineberger Comprehensive Cancer Center, Chapel Hill, NC, USA.
[41] University of North Carolina at Chapel Hill, Department of Epidemiology, Gillings School of Global Public Health and UNC Lineberger Comprehensive Cancer Center, Chapel Hill, NC, USA.
[42] University of Oxford, Nuffield Department of Population Health, Oxford, UK.
[43] Harvard T.H. Chan School of Public Health, Department of Epidemiology, Boston, MA, USA.
[44] Brigham and Women's Hospital and Harvard Medical School, Channing Division of Network Medicine, Department of Medicine, Boston, MA, USA.
[44] Weill Cornell Medicine, Department of Population Health Sciences, New York, NY, USA.
[46] Lunenfeld-Tanenbaum Research Institute of Mount Sinai Hospital, Fred A. Litwin Center for Cancer Genetics, Toronto, ON, Canada.
[47] University of Toronto, Department of Molecular Genetics, Toronto, ON, Canada.
[48] The University of Edinburgh, Usher Institute of Population Health Sciences and Informatics, Edinburgh, UK.
[49] The University of Edinburgh, Cancer Research UK Edinburgh Centre, Edinburgh, UK.
[50] National Cancer Institute, National Institutes of Health, Department of Health and Human Services, Division of Cancer Epidemiology and Genetics, Bethesda, MD, USA.
[51] University of Manchester, Manchester Academic Health Science Centre, Division of Evolution and Genomic Sciences, School of Biological Sciences, Faculty of Biology, Medicine and Health, Manchester, UK.
[52] St Mary’s Hospital, Manchester University NHS Foundation Trust, Manchester Academic Health Science Centre, North West Genomics Laboratory Hub, Manchester Centre for Genomic Medicine, Manchester, UK.
[53] University of Manchester, DivisionÊof Cancer Sciences, Manchester, UK.
[54] Karolinska Institutet, Institute of Environmental Medicine, Stockholm, Sweden.
[55] Uppsala University, Department of Surgical Sciences, Uppsala, Sweden.
[56] University of California Irvine, Department of Medicine, Genetic Epidemiology Research Institute, Irvine, CA, USA.
[57] The Institute of Cancer Research, Division of Genetics and Epidemiology, London, UK.
[58] Queen's University Belfast, Centre for Cancer Research and Cell Biology, Belfast, Ireland, UK.
[59] The Institute of Cancer Research, Division of Breast Cancer Research, London, UK.
[60] National Cancer Institute, Radiation Epidemiology Branch, Division of Cancer Epidemiology and Genetics, Bethesda, MD, USA.
[61] Harvard T.H. Chan School of Public Health, Program in Genetic Epidemiology and Statistical Genetics, Boston, MA, USA.
[62] University Medical Center Hamburg-Eppendorf, Cancer Epidemiology Group, University Cancer Center Hamburg (UCCH), Hamburg, Germany.

Table S1. Demographics characteristics of postmenopausal women in participating studies

Study	Study Design	Cases				Controls			
		N	Age, Mean	Current MHT use	Current EPT use	N	Age, Mean (SD)	Current MHT use	Current EPT use
CBCS	Population-based case-control	379	63.6 (8.1)	0.15	0.06	506	62.8 (8.1)	0.16	0.08
CECILE	Population-based case-control	519	61.2 (7.2)	0.17	0.13	604	61.2 (7.3)	0.14	0.11
CPSII	Prospective cohort	2,362	62.5 (5.8)	0.44	0.20	3,113	62.3 (5.9)	0.39	0.12
EPIC	Prospective cohort	1,680	59.0 (5.5)	0.31	0.26	1,766	58.7 (5.4)	0.31	0.23
GENICA	Population-based case-control	691	62.6 (8.6)	0.27	0.13	533	62.7 (8.7)	0.30	0.12
KARMA	Prospective cohort	836	64.6 (6.7)	0.10	NA	4,153	63.9 (6.2)	0.05	NA
MARIE	Population-based case-control	1,483	63.8 (5.5)	0.44	0.28	1,850	62.7 (5.5)	0.35	0.21
MCBCS	Mixed	973	63.3 (10.2)	0.19	0.19	981	62.1 (9.8)	0.25	0.14
MCCS	Prospective cohort	794	66.6 (8.5)	0.22	NA	874	65.5 (8.1)	0.17	NA
MEC	Prospective cohort	591	64.4 (8.6)	0.59	0.35	618	64.8 (7.7)	0.47	0.21
MISS	Prospective cohort	217	58.4 (6.0)	0.27	NA	591	57.1 (5.2)	0.18	NA
MMHS	Prospective cohort	216	64.6 (8.8)	0.25	0.07	460	63.3 (10.9)	0.28	0.05
NCBCS	Population-based case-control	1,075	60.0 (9.1)	0.25	0.10	596	59.9 (9.0)	0.44	0.15
NHS	Prospective cohort	890	66.6 (8.0)	0.40	0.18	1,513	67.6 (8.4)	0.28	0.10
NHS2	Prospective cohort	508	54.7 (4.4)	0.39	0.23	770	52.7 (5.0)	0.45	0.15
OFBCR	Mixed	666	58.5 (8.2)	0.32	NA	287	57.3 (6.4)	0.52	NA
PBCS	Population-based case-control	1,276	59.9 (8.3)	0.12	0.11	1,390	60.5 (7.4)	0.07	0.05
PKARMA	Mixed	2,320	65.4 (8.3)	0.18	0.05	2,463	60.4 (6.8)	0.08	0.02
PLCO	Prospective cohort	1,819	68.3 (6.4)	0.60	NA	2,578	62.3 (5.2)	0.56	NA
PROCAS	Population-based case-control	244	62.1 (5.4)	0.07	0.04	1,265	61.8 (5.9)	0.07	0.03
SASBAC	Population-based case-control	1,155	63.4 (6.4)	0.37	0.20	1,363	63.3 (6.3)	0.32	0.13
SMC	Prospective cohort	805	61.1 (7.7)	0.27	NA	520	60.8 (7.8)	0.45	NA
UCIBCS	Mixed	259	66.9 (9.7)	0.23	0.07	165	62.6 (7.3)	0.42	0.11
UKBGS	Prospective cohort	648	60.4 (6.9)	0.25	NA	626	60.3 (6.9)	0.17	NA
USRT	Mixed	304	64.1 (8.3)	0.50	NA	639	64.2 (8.5)	0.46	NA
WHI	Prospective cohort	4,875	69.0 (7.4)	0.44	0.25	4,561	69.3 (7.4)	0.36	0.18
**Total**		**27,585**	**64.0 (8.2)**	**0.34**	**0.19**	**34,785**	**63.0 (7.7)**	**0.28**	**0.13**

* CBCS: Canadian Breast Cancer Study; CECILE CECILE Breast Cancer Study; CPSII: Cancer Prevention Study-II Nutrition Cohort; EPIC: European Prospective Investigation Into Cancer and Nutrition; GENICA: Gene Environment Interaction and Breast Cancer in Germany; KARMA: Karolinska Mammography Project for Risk Prediction of Breast Cancer -

Cohort Study; MARIE: Mammary Carcinoma Risk Factor Investigation; MCBCS: Mayo Clinic Breast Cancer Study; MCCS: Melbourne Collaborative Cohort Study; MEC: Multiethnic Cohort; MISS: Melanoma Inquiry of Southern Sweden; MMHS: Mayo Mammography Health Study; NCBCS: North Carolina Breast Cancer Study; NHS: Nurses' Health Study; NHS2: Nurses' Health Study 2; OFBCR: Ontario Familial Breast Cancer Registry; PBCS: NCI Polish Breast Cancer Study; PKARMA: Karolinska Mammography Project for Risk Prediction of Breast Cancer - Case-Control Study; PLCO: The Prostate, Lung, Colorectal and Ovarian Cancer Screening Trial; PROCAS: Predicting the Risk Of Cancer At Screening Study; SASBAC: Singapore and Sweden Breast Cancer Study; SMC: Swedish Mammography Cohort; UCIBCS: UCI Breast Cancer Study; UKBGS: UK Breakthrough Generations Study; USRT: US Radiologic Technologists Study; WHI: Women's Health Initiative Observational Study.

** Current MHT use: Current use of menopausal hormone therapy; Current EPT use: Current use of estrogen + progesterone menopausal hormone therapy

Figure S1. Manhattan plot and quantile-quantile plot of genome-wide interaction of current use of menopausal hormone therapy on breast cancer risk among population-based studies only

A) Manhattan plot

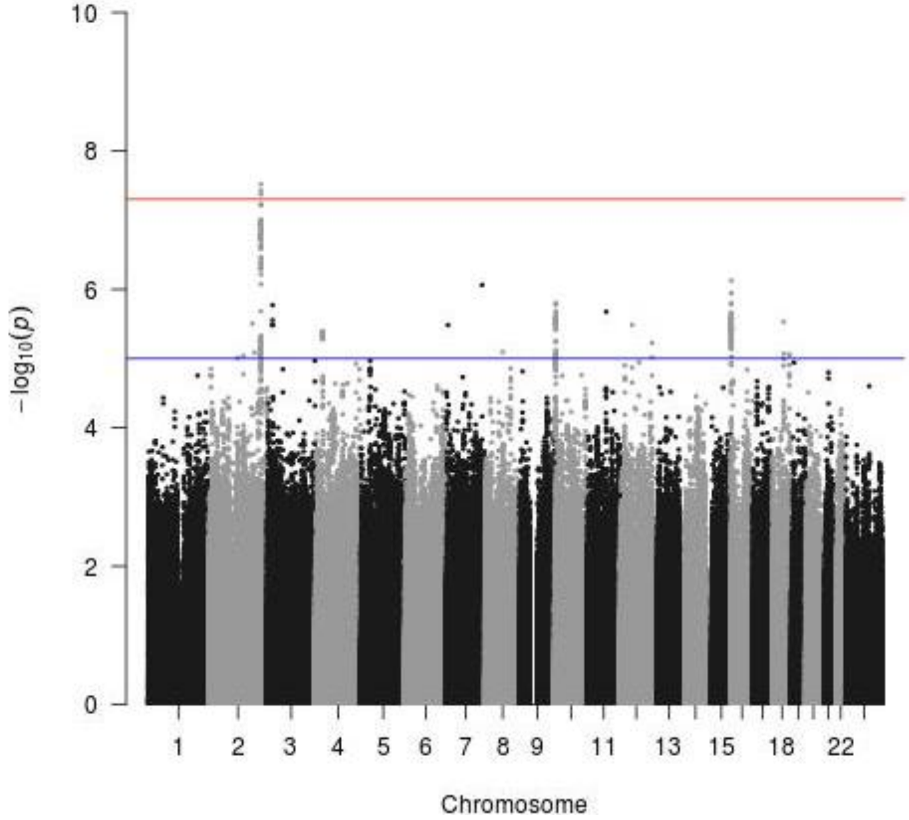

B) Quantile-quantile plot

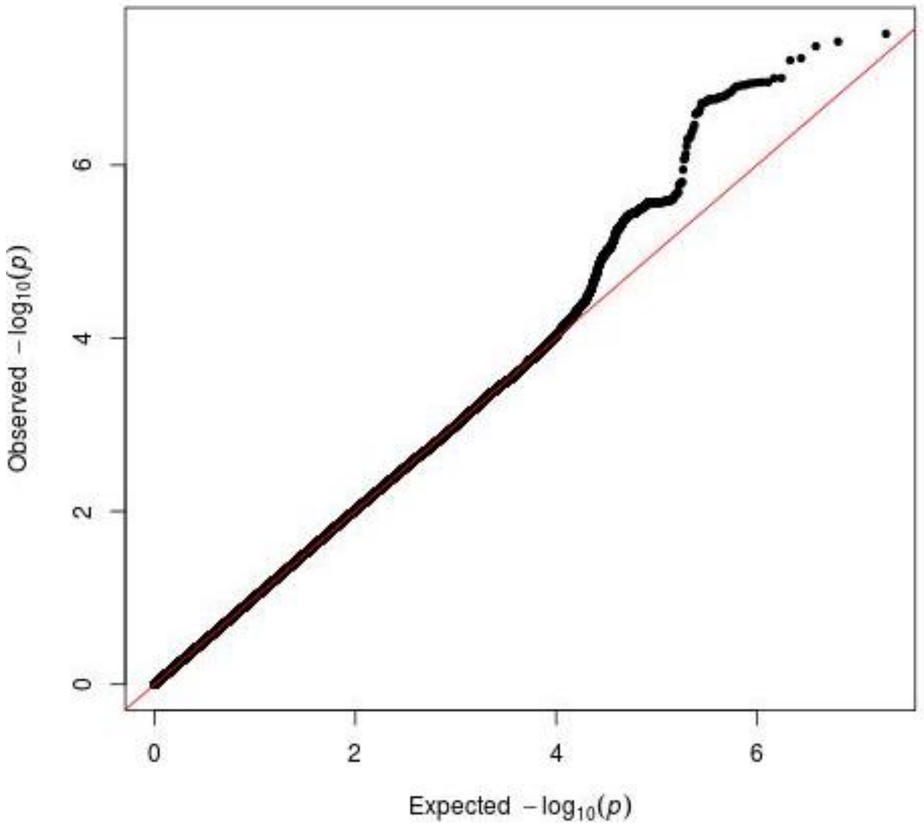

* Red line: log-transformed genome-wide significant threshold at $5x10^{-8}$;
Blue line: log-transformed suggestive threshold at $1x10^{-5}$.

Figure S2. Manhattan plot and quantile-quantile plot of genome-wide interaction of current use of menopausal hormone therapy on estrogen-receptor positive breast cancer risk

A) Manhattan plot

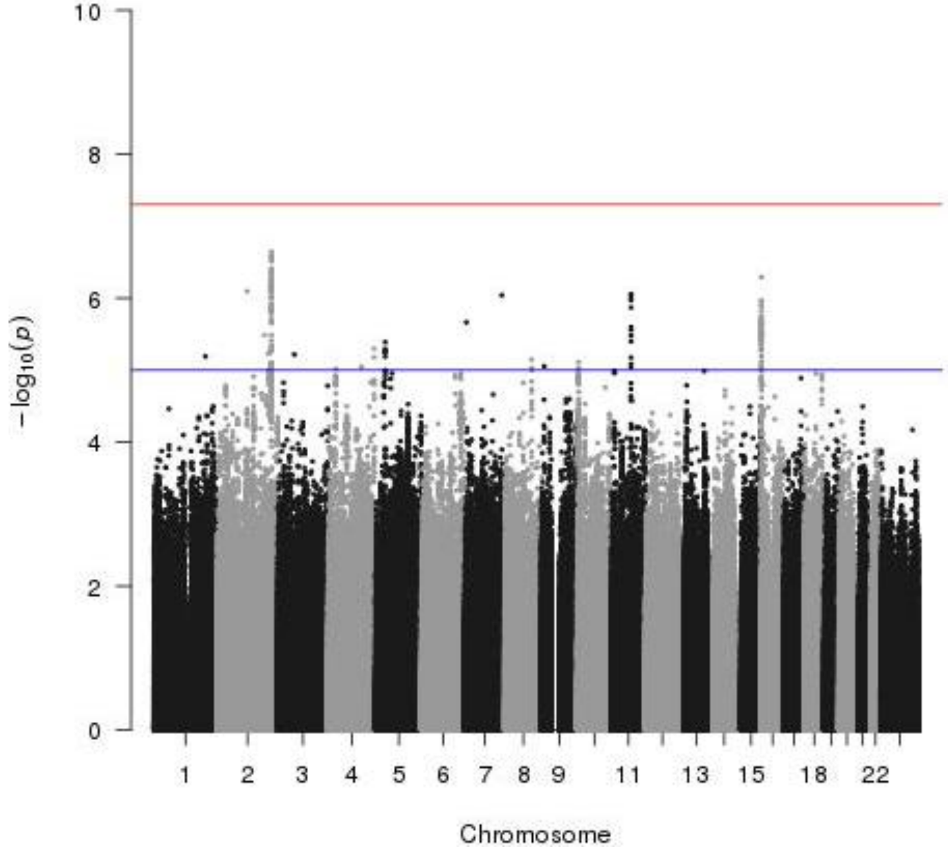

B) Quantile-quantile plot

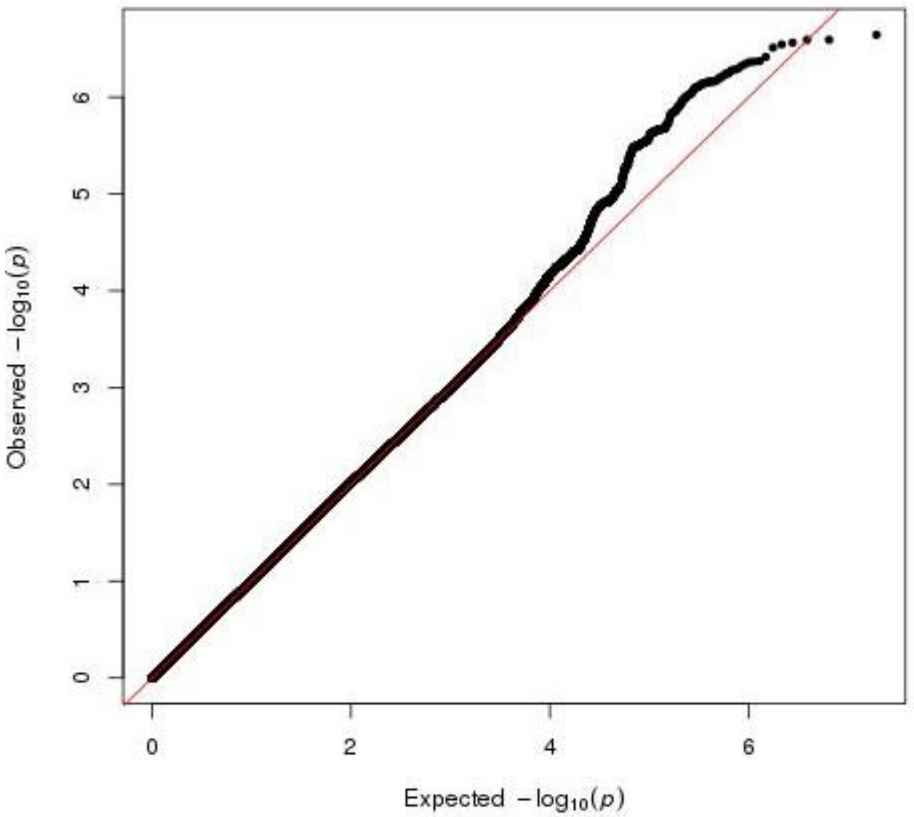

* Red line: log-transformed genome-wide significant threshold at $5x10^{-8}$;
Blue line: log-transformed suggestive threshold at $1x10^{-5}$.

Figure S3. Quantile-quantile plots of genome-wide interaction of current use of menopausal hormone therapy on breast cancer risk

A) Current MHT use

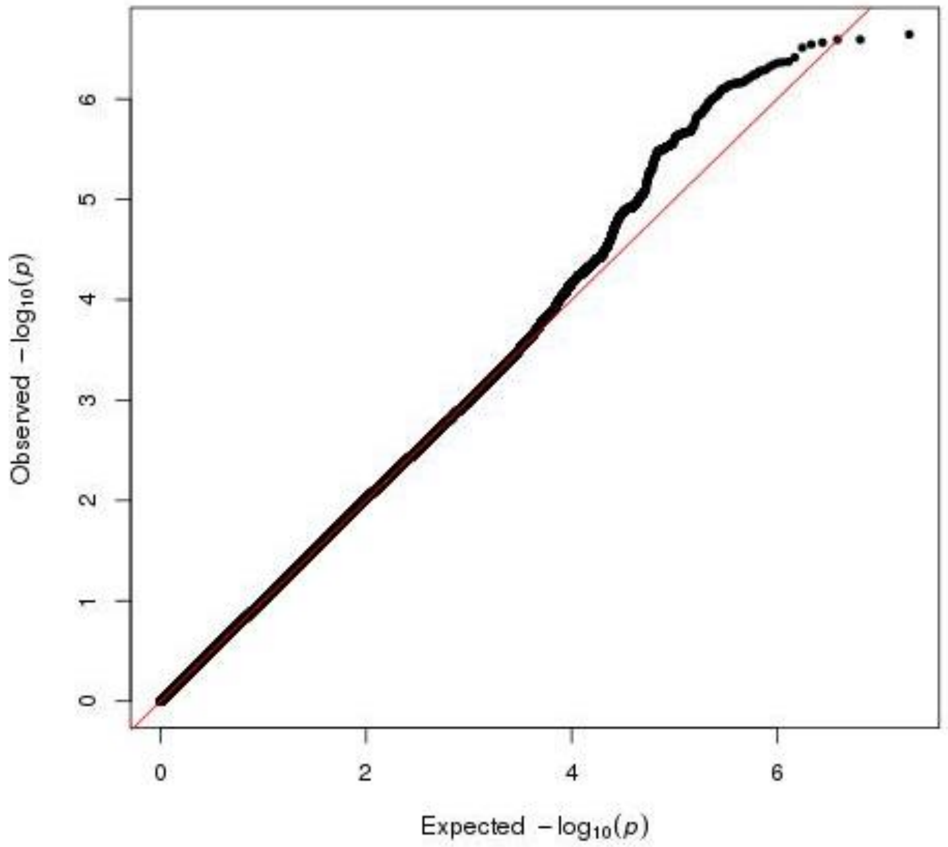

* Genomic control factor λ=1.012

B) Current EPT use

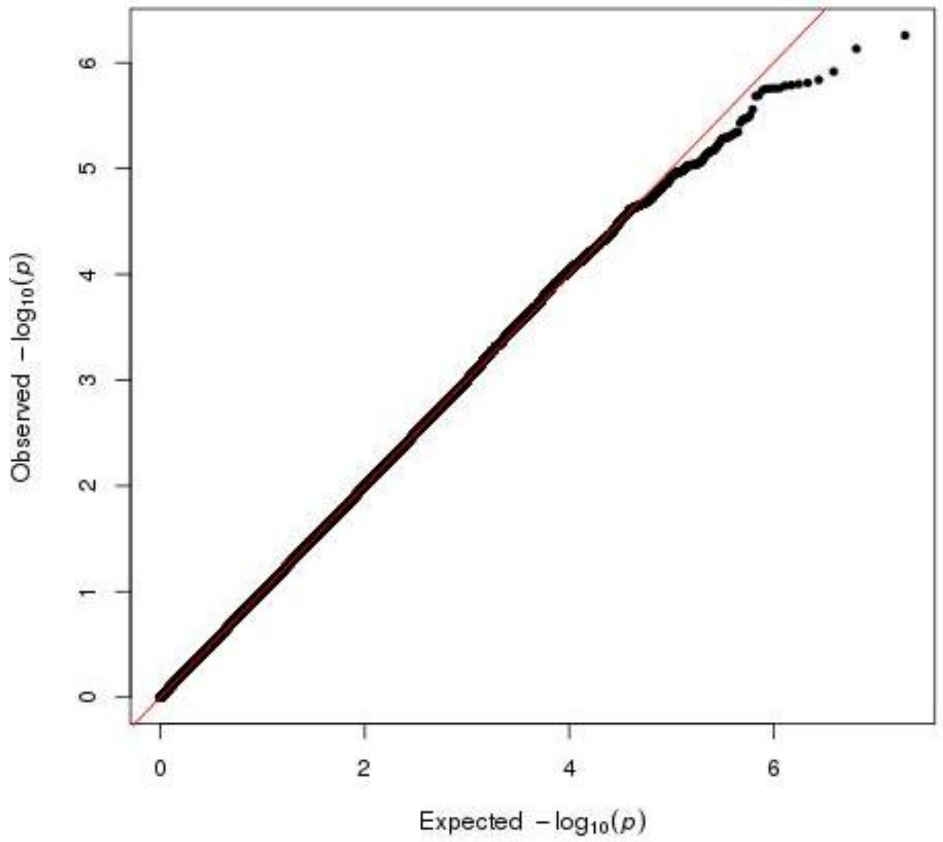

* Genomic control factor λ=1.006
